# Sizing and mending of appendicular muscle mass for hydration during the 12‐lead electrocardiogram: True incidence of sarcopenia in heart failure

**DOI:** 10.1002/jcsm.13423

**Published:** 2024-01-26

**Authors:** Falko Skrabal, Steven B. Heymsfield, Katharina Skrabal, Thomas Weber, Friedrich Fruhwald, Jana Windhaber, Samy Mady

**Affiliations:** ^1^ Institute of Cardiovascular and Metabolic Medicine Graz Austria; ^2^ Pennington Biomedical Research Center Louisiana State University Baton Rouge LA USA; ^3^ Department of Cardiology Klinikum Wels‐Grieskirchen Wels Austria; ^4^ Department of Cardiology Medical University of Graz Graz Austria; ^5^ Department of Paediatric and Adolescent Surgery Medical University Graz Austria

**Keywords:** 12‐channel ECG, Combyn ECG, extracellular water, interstitial, oedema

## Abstract

**Background:**

Our aim was to develop and evaluate a method for the measurement of muscle mass during the 12‐channel electrocardiogram (ECG), to determine the incidence of sarcopenia in patients with overhydration and to correct it for congestion.

**Methods:**

A 12‐channel ECG that simultaneously provided multifrequency segmental impedance data was used to measure total body water (TBW), extracellular water (ECW), ECW/TBW ratio and appendicular muscle mass (AppMM), validated by whole‐body dual‐energy X‐ray absorptiometry. The mean ECW/TBW ratio was 0.24 ± 0.018 (SD) and 0.25 ± 0.016 for young (age range 20–25 years) healthy males (*n* = 77) and females (*n* = 88), respectively. The deviation of the ECW/TBW ratio from this mean was used to correct AppMM for excess ECW (‘dry AppMM’) in 869 healthy controls and in 765 patients with chronic heart failure (CHF) New York Heart Association classes II–IV. The association of AppMM and dry AppMM with grip strength was also examined in 443 controls and patients.

**Results:**

With increasing N‐terminal pro‐brain natriuretic peptide (NT‐proBNP), a continuous decline of AppMM indices is observed, which is more pronounced for dry AppMM indices (for males with NT‐proBNP < 125 pg/mL: AppMM index mean = 8.4 ± 1.05, AppMM index dry mean = 8.0 ± 1.46 [*n* = 201, *P* < 0.001]; for females with NT‐proBNP < 150 pg/mL: AppMM index mean = 6.4 ± 1.0, AppMM index dry mean = 5.8 ± 1.18 [*n* = 198, *P* < 0.001]; for males with NT‐proBNP > 1000 pg/mL: AppMM index mean = 7.6 ± 0.98, AppMM index dry mean = 6.2 ± 1.11 [*n* = 137, *P* < 0.001]; and for females with NT‐proBNP > 1000 pg/mL: AppMM index mean = 5.9 ± 0.96, AppMM index dry mean = 4.8 ± 0.94 [*n* = 109, *P* < 0.001]). The correlation between AppMM and upper‐body AppMM and grip strength (*r*‐value) increased from 0.79 to 0.83 (*P* < 0.001) and from 0.80 to 0.84 (*P* < 0.001), respectively, after correction (*n* = 443). The decline of AppMM with age after correction for ECW is much steeper than appreciated, especially in males: In patients with CHF and sarcopenia, the incidence of sarcopenia may be up to 30% higher after correction for ECW excess according to the European (62% vs. 57%, for males, and 43% vs. 31%, for females) and Foundation for the National Institutes of Health (FNIH) (56% vs. 46%, for males, and 54% vs. 38%, for females) consensus guidelines.

**Conclusions:**

The incidence of sarcopenia in CHF as defined by the European Working Group on Sarcopenia and FNIH consensus may be up to 30% higher after correction for ECW excess. This correction improves the correlation between muscle mass and strength. The presented technology will facilitate, on a large scale, screening for sarcopenia, help identify mechanisms and improve understanding of clinical outcomes.

## Introduction

Despite the fact that muscle strength^1^ and mass are related to prognosis and mortality in many diseases, including chronic heart failure (CHF),^2^, ^3^, ^4^ muscle mass is not routinely determined in internal medicine. Whole‐body dual‐energy X‐ray absorptiometry (DXA) is the standard method to assess appendicular muscle mass (AppMM). However, whole‐body DXA measures water in the extremities, assuming that this water corresponds exclusively to muscle mass. Our hypothesis was that in oedematous states, DXA may overestimate muscle mass due to the accumulation of extracellular fluid. Segmental multifrequency bioimpedance analysis (BIA) is also an alternative approach for measuring AppMM.^5^, ^6^ Its inclusion into the 12‐channel electrocardiogram (ECG)^7^ provides, without time delay, measurement of AppMM and hydration status and important clues for the diagnosis of CHF^7^—and this with a methodology that is performed routinely in internal medicine. We have shown that the measurement of AppMM by segmental multifrequency BIA can give erroneous results in patients with congestion.^7^ We suggested a correction for AppMM congestion (‘dry AppMM’) by simultaneously measuring extracellular water and total body water (ECW and TBW) and deriving the ECW/TBW ratio.^7^ The aim of our study was to evaluate the incidence of sarcopenia corrected for congestion and whether this adjustment improves the agreement between muscle mass and muscle strength.

## Methods

### Participants

**Figure 1:**
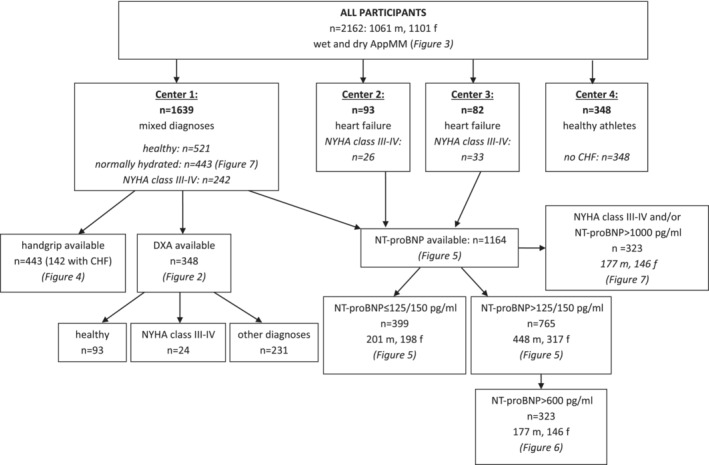
Overview of the participating centres and numbers of patients (*n* = 2162: 1061 males [m] and 1101 females [f]; see *Table* *1* and *Figure* *3*). AppMM, appendicular muscle mass; CHF, chronic heart failure; DXA, dual‐energy X‐ray absorptiometry; NT‐proBNP, N‐terminal pro‐brain natriuretic peptide; NYHA, New York Heart Association.

Our study includes 2162 participants (age range 20–95 years, 1061 males and 1101 females) recruited in four centres. See *Figure* *1* for an overview of all participants from the four centres. Centres 1–3 are heart failure clinics supervised by cardiologists, and Centre 4 is an outpatient unit for sports and exercise. The participants were recruited to cover the complete range of AppMM from athletes to sarcopenia encountered in clinical practice. These included healthy participants, endurance athletes and patients attending the Institute of Cardiovascular and Metabolic Medicine for a health check, training advice or medical advice for hypertension, osteoporosis, hyperlipidaemia, coronary heart disease without CHF, rheumatic and degenerative diseases and cured malignancy. These participants define the range of AppMM in patients without heart failure encountered in clinical practice. The range of muscle mass in the control group therefore includes patients with ideal, normal and reduced muscle mass. In 348 participants (93 healthy controls, 24 patients with CHF New York Heart Association [NYHA] classes III–IV and 231 patients with other diagnoses), AppMM was measured by DXA, and in 443 participants (142 patients with CHF), hand grip strength was also measured (all data only from Centre 1). Participants were defined as controls by lack of symptoms for heart failure, normal Doppler echocardiography and/or N‐terminal pro‐brain natriuretic peptide (NT‐proBNP) measurements below 125 and 150 pg/mL, for males and females, respectively.^8^, ^9^, ^10^, ^11^

In order to ascertain a quantitative relation between the degree of heart failure and ECW overload and AppMM, respectively, 399 participants from Centre 1 (201 males and 198 females; see *Figure* *5*) from the normally hydrated control group in whom a normal Doppler echocardiography and NT‐proBNP measurements below 125 pg/mL in males and 150 pg/mL in females were available (Group I) were compared with 765 patients with CHF NYHA classes II–IV diagnosed according to the European Society of Cardiology (ESC) guidelines^13^ (age range 40–93 years, 448 males and 317 females) attending the participating Centres 1–3. Patients with renal diseases, hepatic diseases, malabsorption and malignancy were excluded from the control group. As echocardiographic measurements were not obtained synchronously with the segmental impedance measurements, synchronously obtained NT‐proBNP values were used for classification of heart failure in *Figures* 5, 6, 7 in the Results section. None of the CHF patients shown in these figures had visible or palpable oedema. These patients had NT‐proBNP levels between 125/150 and 400 pg/mL (Group II, *n* = 324), between 400 and 1000 pg/mL (Group III, *n* = 195) and above 1000 pg/mL (Group IV, *n* = 246).

AppMM of a subgroup of these patients with more marked heart failure (classes III and IV according to the ESC guidelines), and/or with NT‐proBNP above 1000 pg/mL^14^, ^15^ (*n* = 323, 177 males and 146 females; see *Figure* *6* *C,D*), was compared with 513 control participants (257 males and 256 females; see *Figure* *6* *A,B*) in whom CHF was excluded by echocardiography and also by NT‐proBNP lower than 125/150 pg/mL.^8^, ^9^, ^10^, ^11^ This strategy was chosen to ensure a homogenous group of patients. A cut‐off value of 1000 pg/mL additionally ensures at least an NYHA class ≥ II of systolic and diastolic heart failure,^16^, ^17^ even in cases with impaired renal function.^17^

Many patients with NT‐proBNP levels above 1000 pg/mL had more than one diagnosis, and therefore, the number of diagnoses is greater than the number of cases: Out of the 323 patients with CHF NYHA classes III–IV and/or NT‐proBNP above 1000 pg/mL (see *Figures* *6* *C,D* and *7* *C,D*), there were 99 patients with coronary heart disease, 108 patients with hypertensive cardiomyopathy, 56 patients with dilative cardiomyopathy, 62 patients with mitral regurgitation and 18 patients with aortic regurgitation. There were no patients with isolated diastolic heart failure. The patients were studied on their usual medication, which included angiotensin‐converting enzyme (ACE) inhibitors or AT1 receptor blockers, beta‐blockers, loop diuretics and mineralocorticoid receptor antagonists.

### Measurements

#### Segmental multifrequency impedance measurements

The participants rested supine with the upper body elevated at a 30° angle for 10 min. Double electrodes were applied for the 12‐channel Combyn™ ECG, and segmental multifrequency impedance measurements at the thorax, abdomen and extremities were performed simultaneously with the ECG, as reported.^6^ All body compartments are calculated using the equations developed previously.^6^ The Combyn™ ECG beside the 12‐channel ECG provides lean body mass (LBM), trunk fat, ECW, AppMM and dry AppMM corrected for congestion. For the thoracic, abdominal and both leg segments, the relations of ECW to TBW (ECW/TBW ratios) in the specific segments were calculated using impedances at 5 and 400 kHz and specific resistances of ECW and TBW as described in our previous paper.^7^

The percentage of ECW of TBW in young healthy males and females is 24% and 25%, respectively. This is close to the reported values of this ratio in humans^18^ and also in isolated organs (e.g., in the rat heart as measured by magnetic resonance imaging [MRI]^19^). The ratio of ECW/TBW in the leg segments, where the bulk of AppMM is located and where hydrostatic pressure is highest, was calculated for control participants and for patients with CHF. A correction factor of 24/(actual ECW/TBW ratio) and of 25/(actual ECW/TBW ratio) at the legs was used for correction in males and females, respectively. Dry AppMM is defined as AppMM multiplied by the correction factor (24/[actual ECW/TBW ratio] and 25/[actual ECW/TBW ratio]), for males and females, respectively.^6^ In young healthy participants, the correction factor is close to one and the results of wet and dry AppMM are similar (see *Figure* *3*). In congested participants, the ratio is smaller than one and dry AppMM is scaled down accordingly. To derive AppMM index and dry AppMM index, both were divided by height squared (*Figure* *3*).

#### Whole‐body dual‐energy X‐ray absorptiometry measurements

Measurements were performed in patients with suspected osteoporosis and/or sarcopenia and in healthy participants and athletes wishing to determine their muscle mass. Whole‐body DXA (Hologic QDR4500A software Version 12.6) was performed as recommended by the manufacturer to measure AppMM.

#### Measurement of hand grip strength

Maximal hand grip strength was measured using the hydraulic Jamar hand grip dynamometer. Three consecutive measurements were performed each over 5 s with the dominant arm at 90° and the elbow beside the body at 1‐min time interval. The mean of the three readings was used for correlation with AppMM and with AppMM of the upper body.

The study complies with the Declaration of Helsinki. It was approved by the ethics committee of the Medical University Graz (vote numbers EK 27‐419 ex 14/15, 29‐301 ex 16/17, 30‐003 ex 17/18 and 30‐466 ex 17/18) and the ethics committee of the Johannes Kepler University Linz (EK number 1071/2018), and all patients gave written informed consent.

### Statistical methods

Sarcopenia was defined according to the European consensus criteria, which are AppMM index = AppMM/height^2^ < 7.0 kg/m^2^ for males and <5.5 kg/m^2^ for females.^12^ Pearson's correlation coefficients between hand grip strength, AppMM and dry AppMM were calculated.

The Kruskal–Wallis test was used to compare the ECW/TBW ratio, AppMM index and dry AppMM index in different NT‐proBNP groups. For this analysis, 399 patients from the control group without heart failure in whom normal NT‐proBNP measurements were available and 765 patients with different degrees of heart failure (= groups II–IV; *Figure* *5*) were included.

Age, weight, body mass index (BMI), % fat and % trunk fat were compared in patients with CHF with and without low muscle mass. In about half of patients of Centre 1, biochemical variables (C‐reactive protein [CRP], haemoglobin A1C [HbA1C], cholesterol, LDL‐cholesterol and triglycerides, NT‐proBNP and creatinine clearance) were available at the time of muscle mass measurement and were also compared by Student's *t*‐test. *P* values < 0.01 were considered significant.

## Results

An overview of all participants of the different centres is given in *Figure* *1*. The anthropometric data of all participants from the four centres (controls and patients with CHF) are indicated in *Table* *1*. Only Centres 1–3 supplied patients with heart failure, and participants from Centre 4 are represented in *Figure* *3* only.

**Table 1:** Anthropometric data of all male and female participants (controls and patients) of the four different centres (*n* = 2162)

	CHF and controls	
	Males *n* = 1061 Mean ± 2SD^a^	Females *n* = 1101 Mean ± 2SD^a^
Age (years)	60. 2 ± 36.6	56.2 ± 36.0
Weight (kg)	83.1 ± 24.8	67.3 ± 25.4
Height (cm)	177.3 ± 13.4	165.2 ± 13.2
BMI (kg/m^2^)	26.4 ± 7.2	24.7 ± 9.2
Fat (kg)	17.0 ± 14.8	19.5 ± 16.0
Fat trunk (kg)	9.6 ± 9.0	8.1 ± 9.2
Appendicular muscle mass (kg)	26.0 ± 7.8	17.3 ± 6.2
Upper appendicular muscle mass (kg)	6.9 ± 2.2	3.6 ± 1.6
Lower appendicular muscle mass (kg)	19.1 ± 5.8	13.7 ± 4.8
Dry appendicular muscle mass (kg)	24.0 ± 10.2	15.6 ± 7.0
ECW (L)	9.8 ± 2.6	7.2 ± 2.2
TBW (L)	41.0 ± 10.8	28.6 ± 8.8

Abbreviations: BMI, body mass index; CHF, chronic heart failure; ECW, extracellular water; TBW, total body water.

^a^ Corresponding to 95% confidence interval.

**Figure 2:**
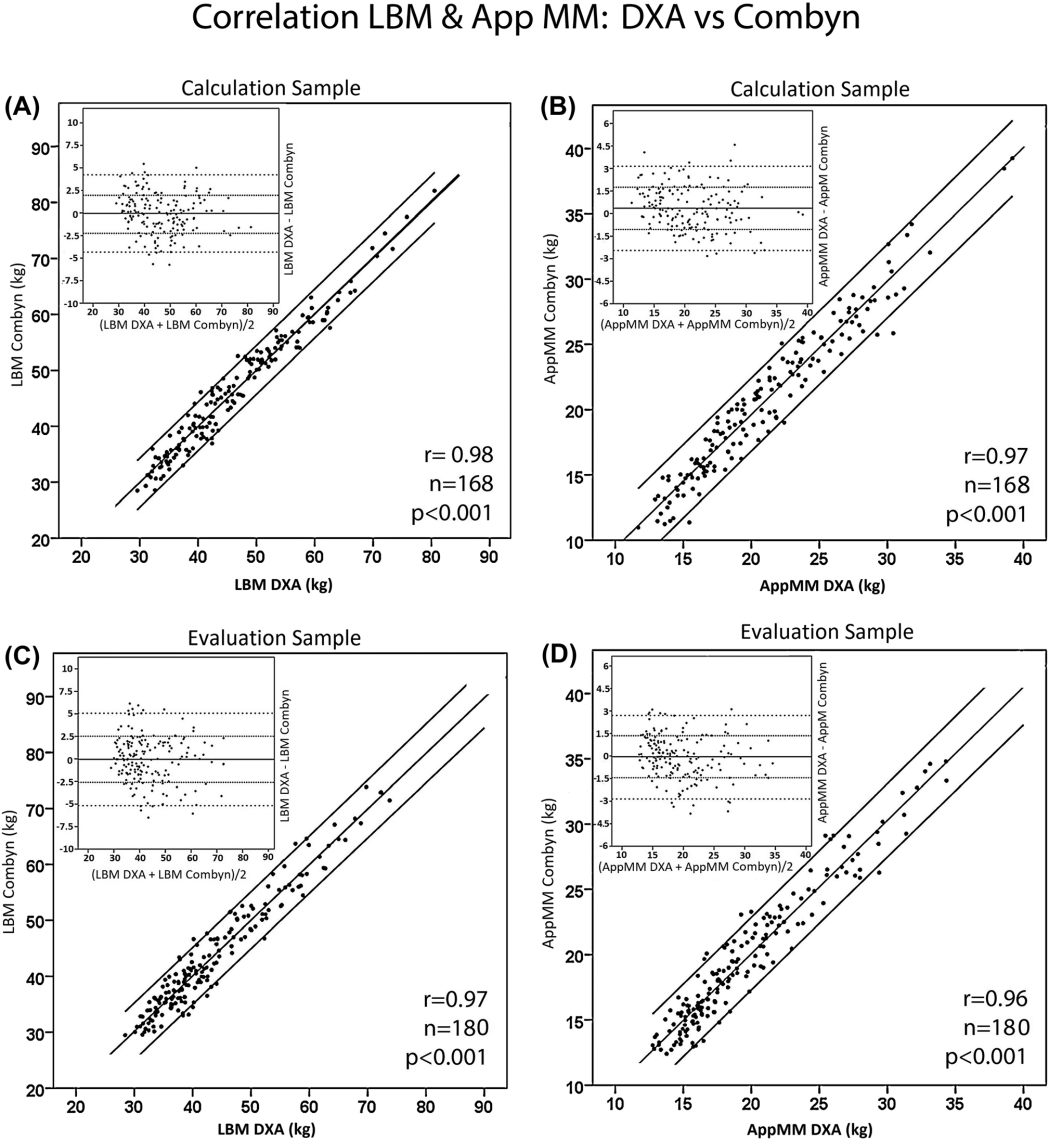
The correlation between lean body mass (LBM) and appendicular muscle mass (AppMM) measured by dual‐energy X‐ray absorptiometry (DXA) and as calculated from the equations included in the software of the Combyn™ ECG is shown in the upper part of the figure (A and B). The lower part of the figure (C and D) shows the relation in the evaluation sample not used for the calculation. The Bland–Altmann plots are shown as inserts (all data from Centre 1).

The correlations between LBM and AppMM, as derived by DXA and as calculated by the equations of the Combyn™ ECG,^7^ are shown in *Figure* *2* *A,B* and *C,D*, respectively. Equations were developed in ~50% of randomized patients (‘calculation sample’) and verified in the other 50% of patients (‘evaluation sample’). The lower part (*2* *C,D*) of the figure shows the relation of these variables in the evaluation sample not used for the calculation. The correlation coefficients and the Bland–Altman plots are shown as inserts (all data from Centre 1).

**Figure 3:**
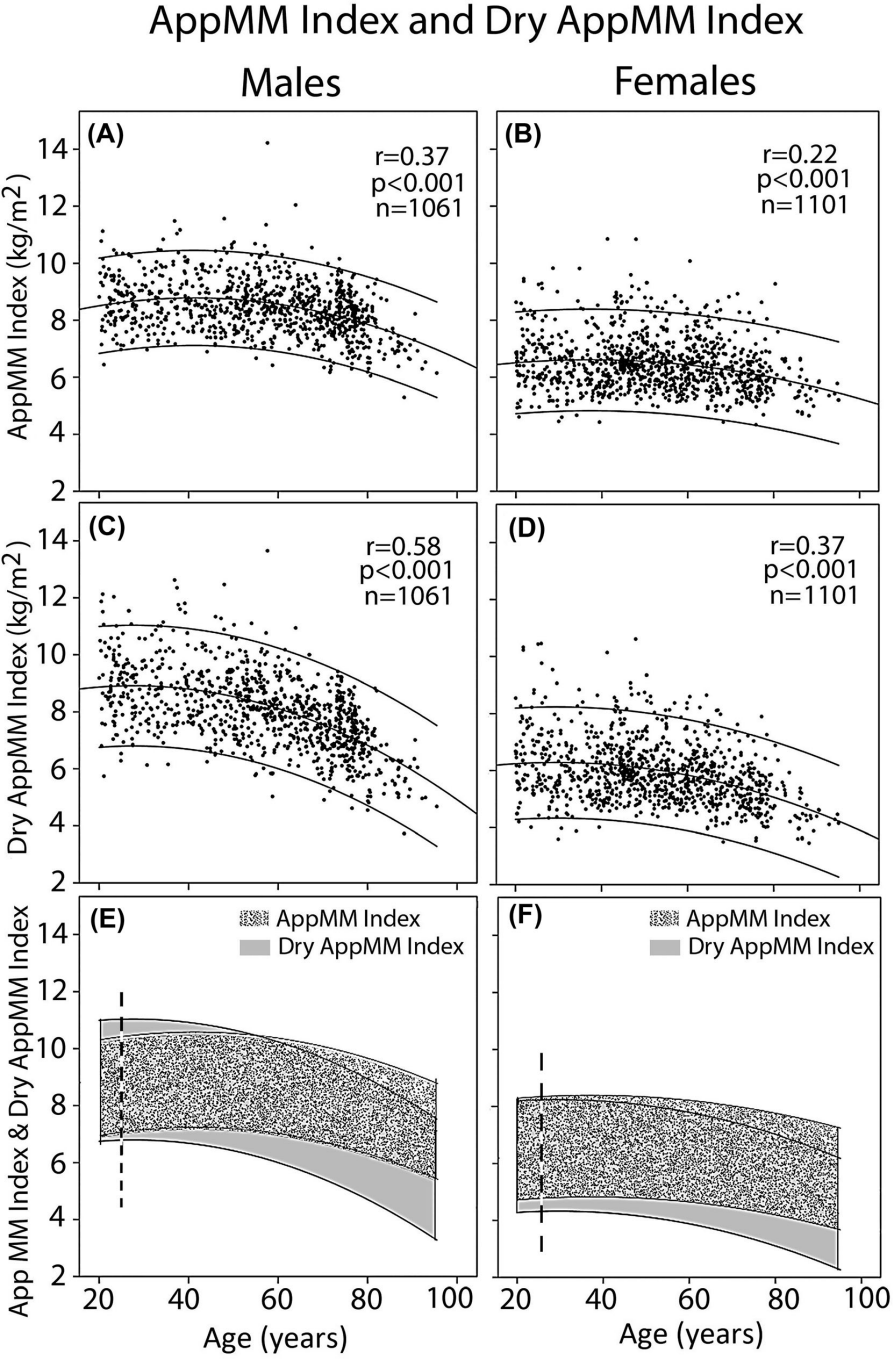
The relation of appendicular muscle mass (AppMM) index (A and B) and dry AppMM index (C and D) in all participants of the four centres, including healthy athletes, patients with chronic heart failure and those with other diagnoses, across the adult life span. Dry AppMM index is higher than AppMM index in young males, but it is lower than AppMM index in females (E and F, left of the dashed line). A steeper decline of dry AppMM index with age is seen in both sexes, implying extracellular water accumulation with age also in the control group.

The range of AppMM index and dry AppMM index in all 2162 participants of all four centres with and without sarcopenia across the adult age range in male (*n* = 1061, left, A, C and E) and female (*n* = 1101, right, B, D and F) participants is shown in *Figure* *3*. In young females (age range 20–25 years), AppMM index is marginally but significantly higher than dry AppMM index (mean = 6.7 ± 1.01 kg/m^2^ vs. mean = 6.4 ± 1.25 kg/m^2^ [*n* = 88, *P* < 0.001]). A progressive fall of AppMM and dry AppMM indices is observed with increasing age, which is more pronounced for dry AppMM index and especially so for male participants (*Figure* *3* *E,F*).

**Figure 4:**
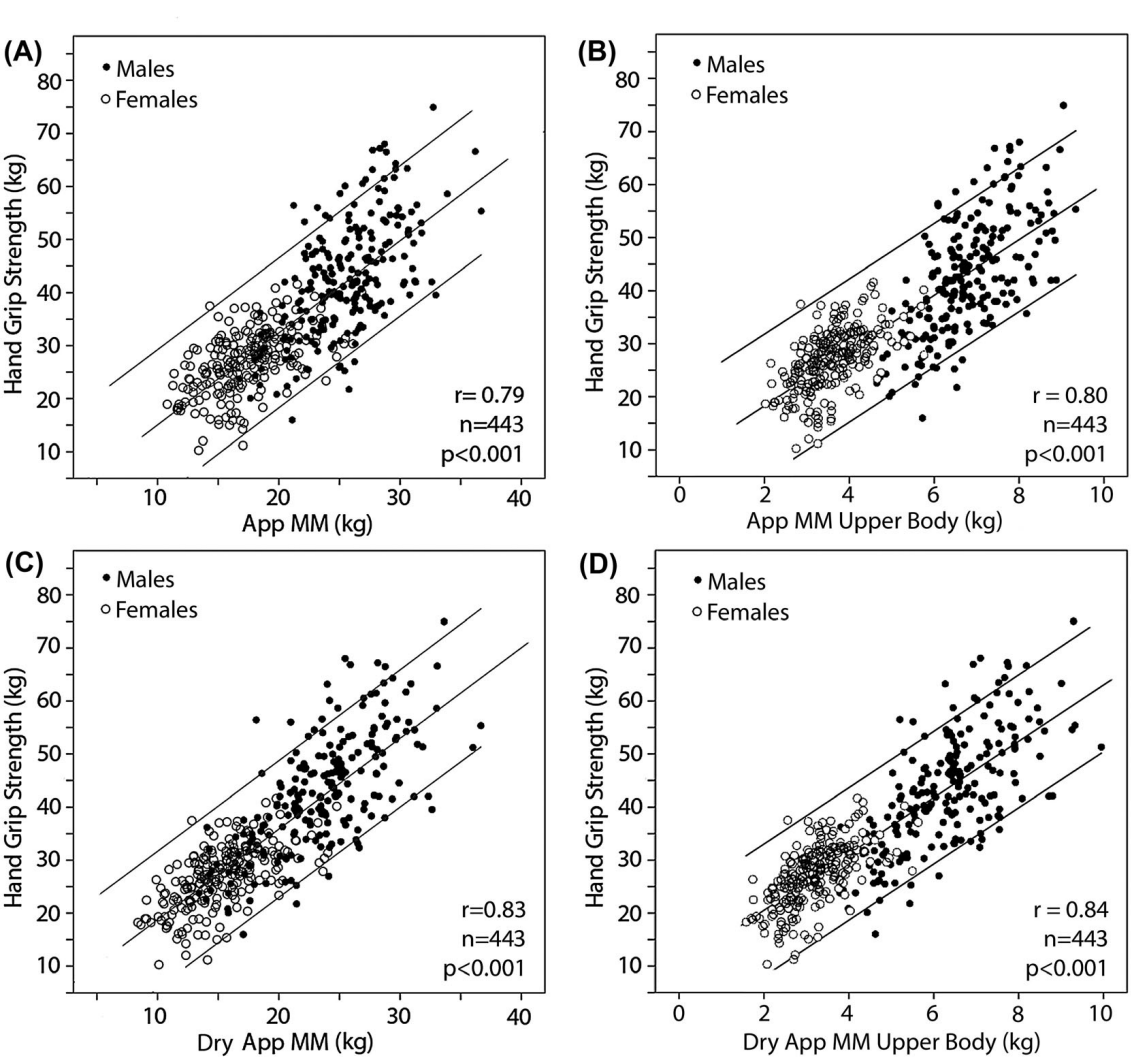
Left panel: Appendicular muscle mass (AppMM) (A) and dry AppMM (C) versus hand grip strength. Right panel: Upper‐body AppMM (B) and upper‐body dry AppMM (D) versus hand grip strength. After correction of AppMM for extracellular water excess, the correlation between AppMM index and upper AppMM index with hand grip strength improves significantly (data from Centre 1).

The correlation between whole‐body (left, A and C) and upper‐body AppMM (right, B and D) and dry AppMM with hand grip strength is shown in *Figure* *4* in the upper (A and B) and lower parts (C and D) of the figure, respectively. The correlation between hand grip strength and AppMM or upper AppMM is significantly better (*P* < 0.001^20^) after correction of AppMM for ECW excess (dry AppMM).

**Figure 5:**
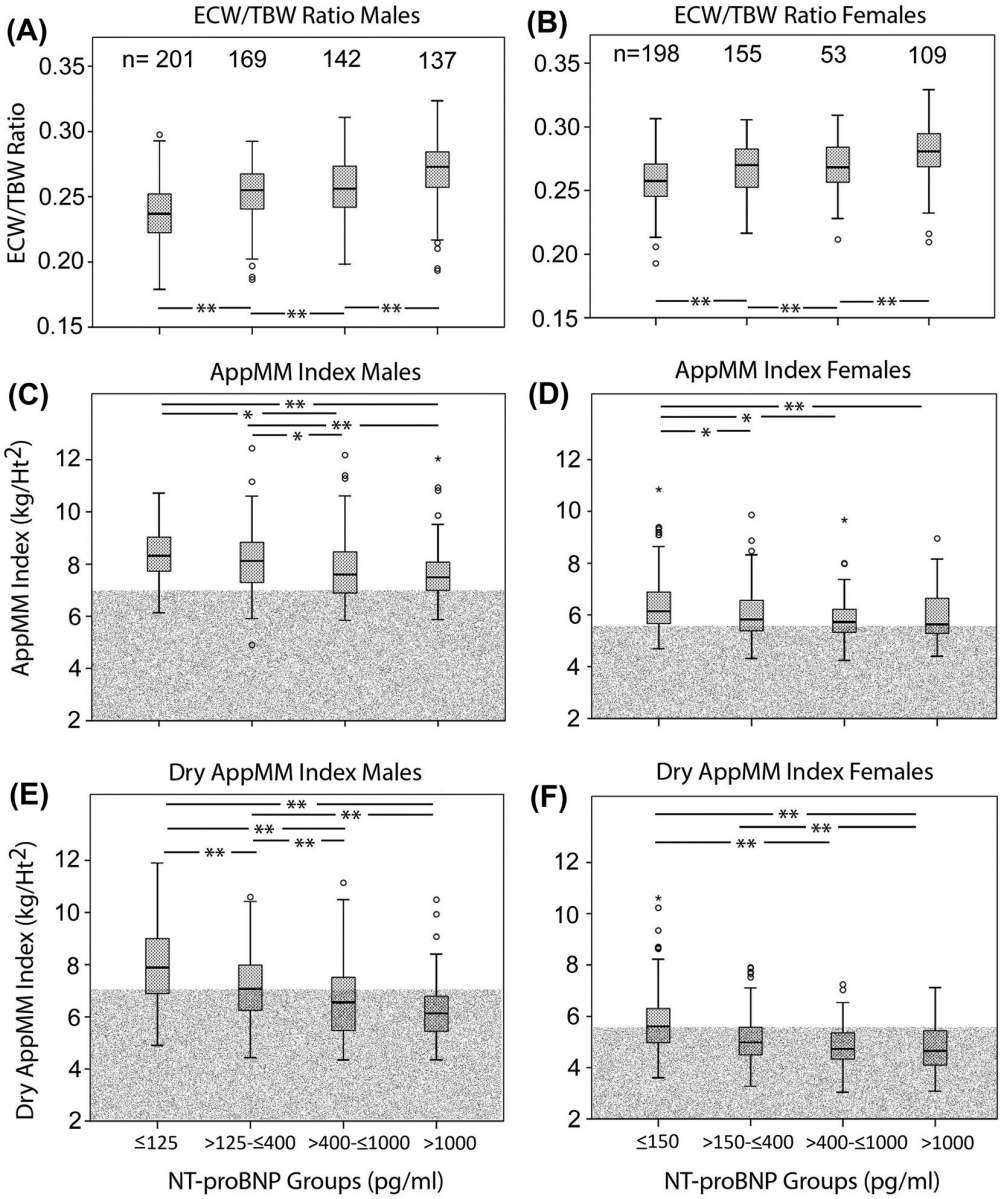
Extracellular water/total body water (ECW/TBW) ratio (A and B), appendicular muscle mass (AppMM) index (C and D) and dry AppMM index (E and F) with different ranges of N‐terminal pro‐brain natriuretic peptide (NT‐proBNP) levels. With increasing NT‐proBNP, there is a significant increase of ECW/TBW ratio and a significant decrease of dry AppMM index (** denotes *P* < 0.001; * denotes *P* < 0.01). The shaded areas correspond to the definitions for sarcopenia^12^ according to the European consensus guidelines for AppMM index. The majority of patients do not have sarcopenia according to the European consensus guidelines if AppMM index is not corrected for ECW excess (data from Centres 1–3).

ECW/TBW ratios of the legs (*Figure* *5*, top panel, A and B), AppMM index (second panel, C and D) and dry AppMM index (bottom panel, E and F) in control participants without heart failure and with normal NT‐proBNP levels and in CHF classified according to different NT‐proBNP levels are shown in *Figure* *5*. With increasing NT‐proBNP, the ECW/TBW ratio increased. AppMM decreased only moderately (second panel, C and D), whereas dry AppMM index decreased more pronounced, from each group to the other. Indeed, analyses using the Kruskal–Wallis test showed a more pronounced difference between AppMM index and dry AppMM index for patients with heart failure as compared with those without: for males with NT‐proBNP < 125 pg/mL: AppMM index mean = 8.4 ± 1.05, AppMM index dry mean = 8.0 ± 1.46 (*n* = 201, *P* < 0.001); for females with NT‐proBNP < 150 pg/mL: AppMM index mean = 6.4 ± 1.01, AppMM index dry mean = 5.8 ± 1.18 (*n* = 198, *P* < 0.001); for males with NT‐proBNP > 1000 pg/mL: AppMM index mean = 7.6 ± 0.98, AppMM index dry mean = 6.2 ± 1.11 (*n* = 137, *P* < 0.001); and for females with NT‐proBNP > 1000 pg/mL: AppMM index mean = 5.9 ± 0.96, AppMM index dry mean = 4.8 ± 0.94 (*n* = 109, *P* < 0.001).

**Figure 6:**
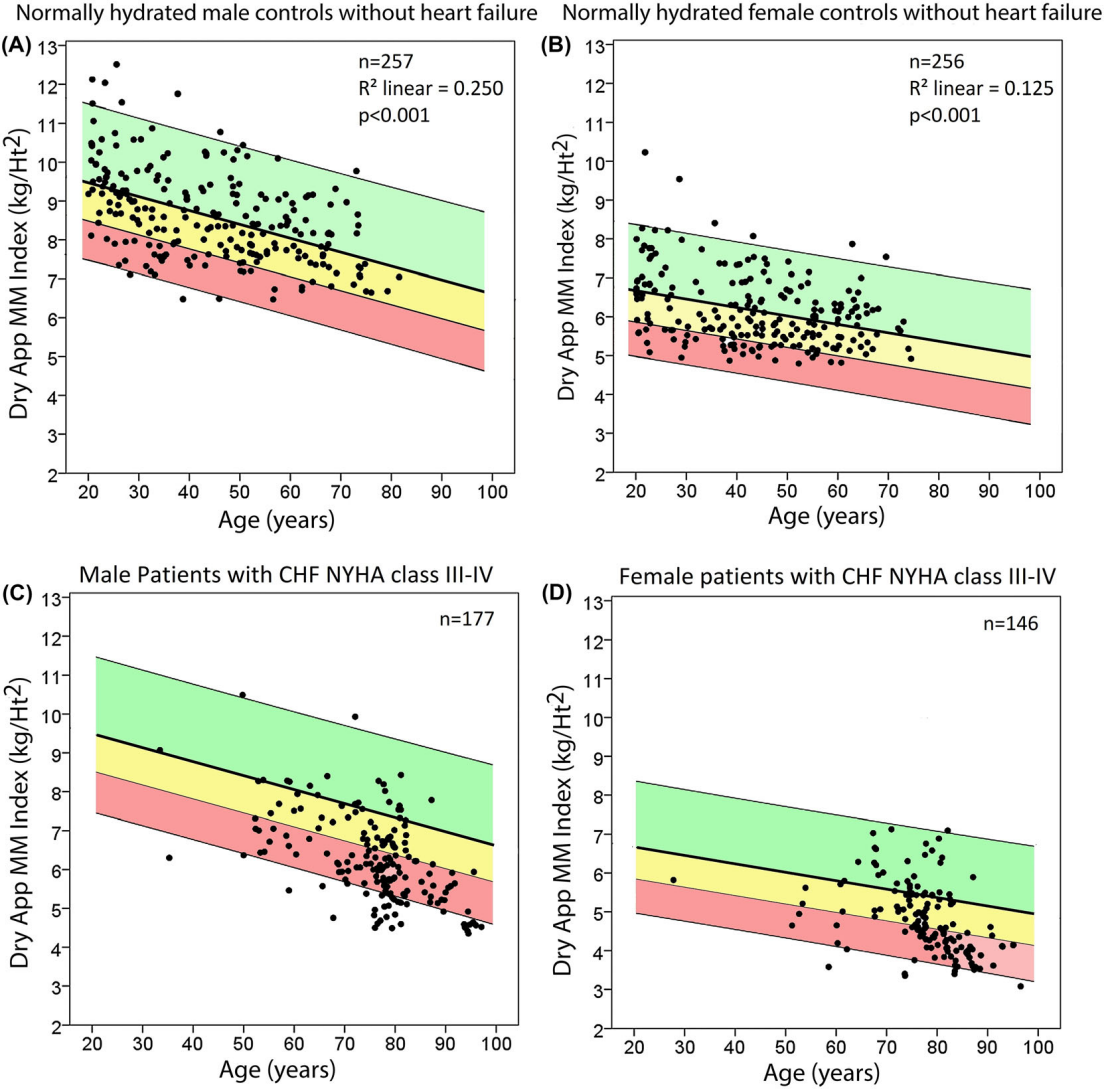
The nomograms with green, yellow and red shaded areas are derived from ‘wet’ appendicular muscle mass (AppMM) indices of healthy, normally hydrated males and females. The line between the yellow and red areas denotes −1SD (*z*‐score −1) below the regression line. When AppMM index in these participants is corrected for hydration (dry AppMM index), the majority of participants lie within the green and yellow areas (mean +2SD and −1SD, Panels A and B). In contrast, the majority of patients with chronic heart failure (CHF) (New York Heart Association [NYHA] classes III–IV and/or N‐terminal pro‐brain natriuretic peptide > 600 pg/mL, lower part of the figure, C and D) lie within the red area (lower than mean −1SD), showing a high incidence of low muscle mass (data from Centres 1–3).

The dry AppMM indices in male and female participants with CHF NYHA class III and IV and/or NT‐proBNP levels above 600 pg/mL, in relation to the range of ‘wet’ AppMM indices observed in the normally hydrated control group without heart failure, are shown in *Figure* *6* (A and C for males and B and D for females, respectively). Patients with renal diseases, hepatic diseases, malabsorption and malignancy were excluded from the control group. Also, none of the CHF patients shown in this figure had visible or palpable oedema. When AppMM index in the control group of healthy, normally hydrated participants is corrected for hydration (dry AppMM index), the majority of participants lie within the green and yellow area (mean +2SD and −1SD, Panels A and B). In contrast, the majority of patients with CHF (NYHA classes III–IV and/or NT‐proBNP > 600 pg/mL, lower part of the figure, C and D) lie within the red area (lower than mean −1SD; *z*‐score < −1), showing a high incidence of low muscle mass. The percentage of heart failure patients with sarcopenia as defined by the European consensus^12^ increased in males and females when dry (as opposed to ‘wet’) AppMM index was used (57% [*n* = 346] vs. 62% [*n* = 378] in males and 31% [*n* = 150] vs. 43% [*n* = 207] in females). Likewise, according to the FNIH consensus that takes into account grip strength and appendicular LBM adjusted for BMI,^21^ the percentage increased from 46% (*n* = 283) to 56% (*n* = 341) in males and from 38% (*n* = 186) to 54% (*n* = 259) in females.

**Figure 7:**
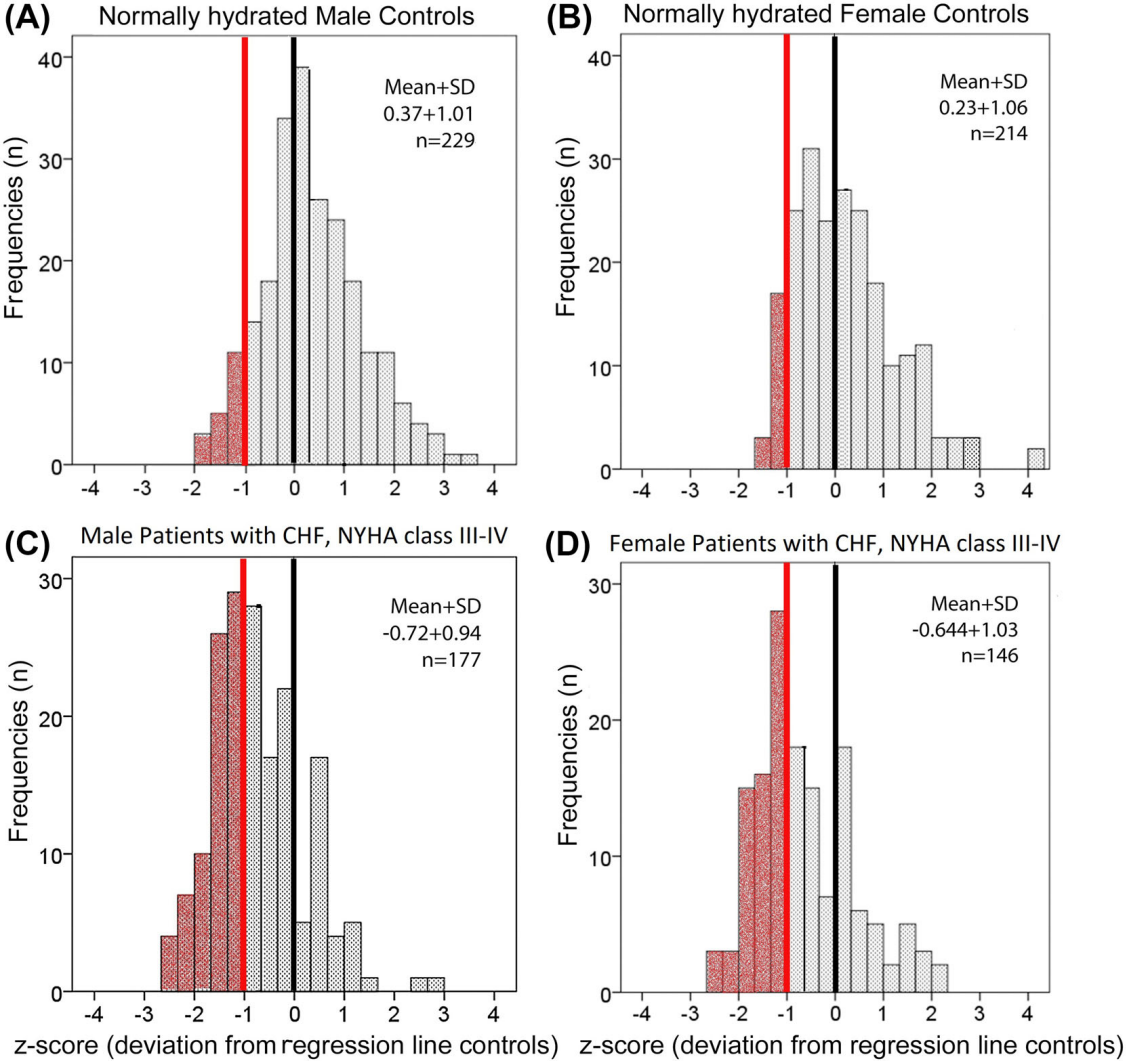
Histograms of *z*‐scores in males (A and C) and females (B and D). Normally hydrated participants (*n* = 443) are shown on the upper panel (A and B), and patients with chronic heart failure (CHF) (New York Heart Association [NYHA] classes III–IV and/or N‐terminal pro‐brain natriuretic peptide > 600 pg/mL, *n* = 323) are shown on the bottom panel (C and D). *Z*‐scores are shifted to lower values in patients with CHF (data from Centres 1–3).

This increase of percentage of low muscle mass due to the correction of AppMM index for hydration is shown in the histogram in *Figure* *7* as the deviation of AppMM index from the regression line obtained from normally hydrated controls without heart failure.

When patients with and without low muscle mass were compared (*z*‐score dry AppMM of < −1 vs. > −1), age and % body fat were comparable, but patients with low muscle mass had lower BMI (24.2 vs. 28.6, *P* < 0.001) and trunk fat % body weight (12.8 vs. 14.6%, *P* < 0.001) and higher log NT‐proBNP (3.27 vs. 3.11 pg/mL, *P* < 0.007). Creatinine clearances in patients with low muscle mass were lower (48.6 vs. 55.1 mL/min, *P* < 0.002) as compared with patients with normal muscle mass (data only available from Centre 1). CRP, HbA1C, cholesterol, LDL‐cholesterol and triglycerides were comparable for both groups (data not shown).

## Discussion

Measurements of AppMM as part of a routine investigation in patients attending medical help could improve patient health care considerably. Therefore, we have included this technology into the routine 12‐channel ECG, which is performed in the majority of patients seeking medical advice. Especially in the older part of the population, a therapeutic strategy based on these measurements could help to counteract frailty and falls and could prolong active live, reducing the cost of health care. This study shows that the incidence of sarcopenia in CHF is higher than appreciated if its measurement is corrected for congestion, whereas measurements of AppMM uncorrected for ECW excess misclassify many cases because of subclinical overhydration.^6^ So far, only poor correlations between muscle mass and function have been reported,^22^, ^23^, ^24^ correlation coefficients ranging between 0.49 and 0.66.^25^ Our results indicate that our methodology provides a better estimate of AppMM than previous studies using DXA or segmental multifrequency impedance. We consider it an important advantage that the extremity electrodes are applied above the ankles and wrists, where the distribution of fat, water and muscle shows representative physiological proportions and where the hands and feet, consisting mainly of connective tissue, are excluded.^26^, ^27^ The impedances of hand and feet do not contribute to the compartments of interest and cause the major and a variable part of the series resistance that can falsify the resistances of interest in an unpredictable way. A further advantage might be that we analyse a six‐segment model, consisting of extremities and the thorax and abdomen separately. This separation seems justified in the light of the completely different composition of the thoracic and abdominal organs. After correcting the AppMM for congestion, an even better correlation between muscle mass and function is observed (*Figure* *4*), indicating that this correction is meaningful.^28^, ^29^ This is further evidence that the measurement of AppMM (e.g., by whole‐body DXA, considered as a gold standard method^30^) is frequently hampered by unnoticed ECW excess.^31^ It is interesting to note that dry AppMM index is lower than ‘wet’ AppMM index in young females, implying that they have already an increased ECW as compared with males, which probably is a result of oestrogens.^32^ The progressive increase of ECW/TBW ratio in both sexes with age may be the result of not only accumulation of ECW but also muscle loss itself with relative increase of fat mass.^33^ As the Combyn™ ECG also enables the measurement of ECW calibrated by bromide dilution,^34^ we were able to show that with increasing severity of CHF, the ratio ECW/TBW increases (*Figure* *5*, upper panel). This and the improvement of the relation between dry AppMM and muscle strength (*Figure* *4*) are arguments for a major contribution of ECW excess to wet AppMM. The present European consensus for defining sarcopenia is a cut‐off value of AppMM/height^2^ of 7.0 kg/m^2^ for males and 5.5 kg/m^2^ for females.^31^ The percentage of patients with sarcopenia increases in males from 57% to 62% and in females from 31% to 43% when using wet and dry AppMM, respectively. The correlation between LBM as measured by DXA and as calculated by the Combyn™ ECG is also excellent (*Figure* *2*, left, A and C). Using the FNIH consensus based on appendicular LBM, the percentage of sarcopenia in males and females with CHF NYHA classes III and IV increased from 46% to 56% and from 38% to 54%, respectively, when using dry instead of wet appendicular LBM.

It would seem logical to define in the future a low muscle mass according to the deviation from the age‐corrected *z*‐score instead of using a fixed value. A *z*‐score of AppMM index < −1 defining a low muscle mass index for age (*Figure* *6*, border between the yellow and red areas) could warn individuals to improve their muscle status before they reach the critical grade of sarcopenia with increasing age. This has been made possible by the large body of data supplied by the methodology presented here. Also, in the future, the current cut‐off for sarcopenia^13^ will have to be adjusted for dry AppMM.

CHF patients with and without sarcopenia had similar age, body fat, inflammation markers, HbA1C and lipids, but CHF patients with sarcopenia according to the current European guidelines had higher NT‐proBNP levels and lower BMI, % trunk fat and creatinine clearance, suggesting more advanced heart failure, reduced kidney function and more general wasting. However, these biochemical data were only available in about half of the patients.

Weaknesses of our study are that the cut‐off for sarcopenia according to dry AppMM index will have to be defined and that prospective studies of dry AppMM on mortality are needed. Using a *z*‐score of AppMM index < −1 could be a useful beginning (*Figure* *7*).

The detection of dry (true) muscle mass is especially relevant in patients in whom sarcopenia and accumulation of extracellular fluid are likely to be concomitant (e.g., in CHF as shown here), but also in chronic renal failure,^35^ in chronic liver disease,^29^ in inflammatory diseases^36^ or in cancer.^37^ In all these conditions, the degree of sarcopenia determines morbidity and mortality. As the measurement of dry AppMM is performed piggyback as a background measurement without time delay during the routine ECG, this could help to provide more attention to the detection of sarcopenia in clinical routine in the future. Prospective studies of dry AppMM on cardiac mortality are needed.

## Funding information

This work was supported by the SFG, Austria (1.000.034.146 and 1.000.037.669) and the FFG, Austria (849750 and 855562).

## Permissions information

The authors do hereby declare that all illustrations and figures in the manuscript are entirely original and do not require reprint permission.

## Conflict of interest statement

All authors declare that there is no conflict of interest.
